# Involvement of resistin-like molecule β in the development of methionine-choline deficient diet-induced non-alcoholic steatohepatitis in mice

**DOI:** 10.1038/srep20157

**Published:** 2016-01-28

**Authors:** Hirofumi Okubo, Akifumi Kushiyama, Hideyuki Sakoda, Yusuke Nakatsu, Masaki Iizuka, Naoyuki Taki, Midori Fujishiro, Toshiaki Fukushima, Hideaki Kamata, Akiko Nagamachi, Toshiya Inaba, Fusanori Nishimura, Hideki Katagiri, Takashi Asahara, Yasuto Yoshida, Osamu Chonan, Jeffery Encinas, Tomoichiro Asano

**Affiliations:** 1 Department of Medical Science, Graduate School of Medicine, University of Hiroshima, 1-2-3 Kasumi, Minami-ku, Hiroshima City, Hiroshima Japan; 2 Institute for Adult Disease, Asahi Life Foundation, 2-2-6, Bakuro-cho, Chuo-ku, Tokyo 103-0002, Japan; 3 Department of Internal Medicine, Graduate School of Medicine, University of Tokyo, 7-3-1 Hongo, Bunkyo-ku, Tokyo; 4 Department of Molecular and Cellular Biology, Kobe Pharma Research Institute, Nippon Boehringer-Ingelheim Co. Ltd. 6-7-5, Minatoshimaminami-cho, Chuo-ku, Kobe city, Hyogo 650-0047, Japan; 5 Department of Molecular Oncology and Leukemia Program Project, Research Institute for Radiation Biology and Medicine, Hiroshima University, Hiroshima 734-8553, Japan; 6 Department of Dental Science for Health Promotion, Division of Cervico-Gnathostomatology, Graduate School of Biomedical Sciences, Hiroshima University, 2-1 Seiryo-machi, Aoba-ku, Sendai, Japan; 7 Division of Molecular Metabolism and Diabetes, Tohoku University Graduate School of Medicine, 2-1 Seiryo-machi, Aoba-ku, Sendai, Japan; 8 Yakult Central Institute, Yakult Honsha Co., Ltd. 5-11, Izumi, Kunitachi-shi, 186-8650 Tokyo, Japan

## Abstract

Resistin-like molecule β (RELMβ) reportedly has multiple functions including local immune responses in the gut. In this study, we investigated the possible contribution of RELMβ to non-alcoholic steatohepatitis (NASH) development. First, RELMβ knock-out (KO) mice were shown to be resistant to methionine-choline deficient (MCD) diet-induced NASH development. Since it was newly revealed that Kupffer cells in the liver express RELMβ and that RELMβ expression levels in the colon and the numbers of RELMβ-positive Kupffer cells were both increased in this model, we carried out further experiments using radiation chimeras between wild-type and RELMβ-KO mice to distinguish between the contributions of RELMβ in these two organs. These experiments revealed the requirement of RELMβ in both organs for full manifestation of NASH, while deletion of each one alone attenuated the development of NASH with reduced serum lipopolysaccharide (LPS) levels. The higher proportion of lactic acid bacteria in the gut microbiota of RELMβ-KO than in that of wild-type mice may be one of the mechanisms underlying the lower serum LPS level the former. These data suggest the contribution of increases in RELMβ in the gut and Kupffer cells to NASH development, raising the possibility of RELMβ being a novel therapeutic target for NASH.

This study aimed to investigate the contribution of resistin like molecule (RELM) β (FIZZ2, mXCP3, hXCP2) to the pathogenesis of non-alcoholic steatohepatitis (NASH) development. NASH is a serious liver disorder, which develops due to hepatic steatosis and then progresses to fibrosis, cirrhosis and finally hepatocellular carcinoma. At present, the “second hit theory” is the mostly widely accepted hypothesis for the molecular mechanism underlying NASH development. The first hit involves simple steatosis, which arises from an excess supply of fatty acids and/or glucose, lipotoxicity, and insulin resistance. The second hit involves aggravating factors such as oxidative stress, inflammatory cytokines and endotoxins that are considered to play important roles as the predominant causes of liver neutrophil infiltration^1^, and the resultant liver damage^2^,^3^. Serum lipopolysaccharide (LPS) elevation appears to function as a trigger of hepatic inflammation, and its continuous infusion reportedly induces hepatosteatosis in mice, suggesting the importance of serum LPS in the pathogenesis of NASH. Furthermore, several recent reports have shown impaired gut functions such as gut hyper-permeability and/or small intestinal bacterial overgrowth to be more frequent in NASH patients than in healthy subjects^4^,^5^,^6^.

On the other hand, RELM β is a protein homologous to resistin, initially identified as a factor secreted by mouse adipocytes which causes insulin resistance^7^. RELMβ has been identified in the digestive and bronchial tracts^8^,^9^, while our recent report revealed abundant expression of RELMβ in the foam cells of atherosclerotic lesions^10^. RELMβ reportedly contributes to local immune system function in the gut and bronchi by acting against bacteria and parasitic nematodes^11^,^12^,^13^. RELMβ is also likely to be one of the factors regulating gut microbiota, since RELMβ absence influences the microbiome composition^14^. Interestingly, RELMβ expression is undetectably low in the colons of germ-free immunocompetent mice^11^. Thus, RELMβ and gut microbiota appear to affect each other, influences which would both contribute to the maintenance of homeostasis including immune and inflammatory responses in the gut.

To date, associations of resistin or RELMβ with several pathological conditions, including insulin resistance^15^, coronary artery disease^16^, congestive heart failure^17^ and intestinal inflammation^18^,^19^,^20^,^21^,^22^, have been suggested. Impaired glucose and lipid metabolism accompanying insulin resistance were reported in mice injected with recombinant RELMβ^15^ as well as transgenic mice overexpressing RELMβ^23^. In addition, RELMβ augments interferon (IFN) γ-induced tumor necrosis factor (TNF) α secretion in thioglycollate-isolated macrophages and infection-induced intestinal inflammation^18^. Dextran sodium sulfate-induced colitis was significantly suppressed in RELMβ knock-out (KO) mice^19^.

In this study, it was clearly demonstrated that RELMβ-KO mice are highly resistant to the development of NASH. During our investigation focusing on RELMβ, unexpectedly, we found that considerable percentages of Kupffer cells in the liver express RELMβ. Thus, to distinguish the roles of RELMβ secreted from the gut versus that from Kupffer cells, radiation chimeras between RELMβ-KO and wild-type mice were prepared. This study provides the first evidence of the critical role of RELMβ in NASH development, and raises the possibility of RELMβ being a target for novel NASH therapies.

## Results

### Development of NASH was suppressed in RELMβ–KO mice

**Figure 1:**
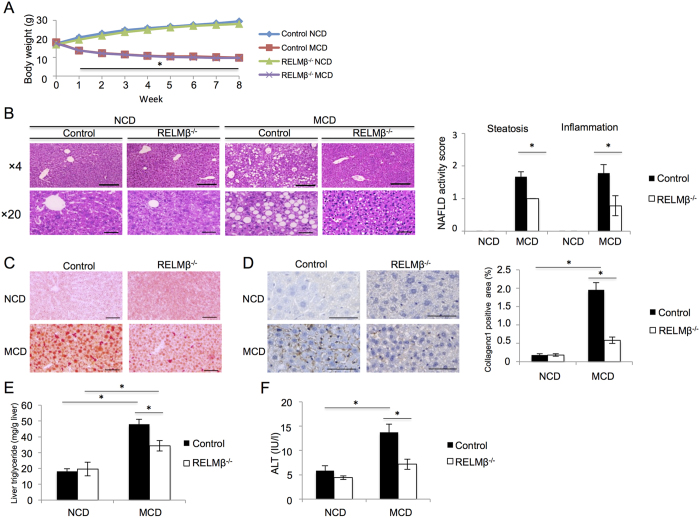
NASH development was suppressed in RELMβ-KO mice. RELMβ-KO and wild-type mice were fed the NCD (n = 6 per strain) or the MCD diet (n = 6 per strain) for 8 weeks and then sacrificed. (**A**) Body weight changes. (**B**) Liver sections were stained with HE. Scale bar = 200 μm (×4 magnification) or 50 μm (×20 magnification). NAFLD activity scores were determined for each mouse. (**C**) Liver sections were stained with Oil-Red O. Scale bar = 50 μm (×20 magnification). (**D**) Immunohistochemical staining for Collagen α-1. Scale bar = 50 μm (×40 magnification). Positively stained areas were counted employing NIH image. (**E**) Hepatic triglyceride levels were measured. (**F**) Serum ALT levels were measured. Data are presented as means ± SE. *Statistical significance P < 0.05.

To investigate the effect of RELMβ on the pathogenesis of NASH, RELMβ-KO and wild-type mice were fed the normal chow diet (NCD) or the methionine-choline deficient (MCD) diet for 8 weeks. Mice fed the MCD diet showed a significant decrease in body weight as compared with those fed the NCD diet. There were no significant differences in body weight between the RELMβ-KO and wild-type mice with either diet (Fig. 1A). The livers were harvested and subjected to histological analysis. Hematoxylin and eosin (HE) staining revealed marked increases in balloon-like structures and deformity of hepatocytes and increased inflammatory cell infiltration in the livers of wild-type mice fed the MCD diet, while these abnormalities were suppressed in RELMβ-KO (Fig. 1B). In addition, Oil-Red-O staining showed highly advanced lipid accumulation in the livers of MCD diet fed wild-type mice, while such accumulation was minimal in RELMβ-KO mice (Fig. 1C). Immunostaining analysis revealed a remarkable accumulation of collagen α1 in the livers of MCD-diet fed wild-type mice. This accumulation is the final step in the development of NASH and is not observed in the livers of animals with simple fatty liver, nor does it occur in those of RELMβ-KO mice (Fig. 1D). The results of these histological analyses were supported by the hepatic triglyceride content data (Fig. 1E) as well as the levels of serum alanine aminotransferase (ALT), an indicator of liver injury (Fig. 1F). This data series suggested RELMβ deficiency to markedly attenuate the development of MCD diet-induced NASH.

### Gene expressions involved in the pathogenesis of NASH were suppressed in the livers of RELMβ-KO mice

**Figure 2:**
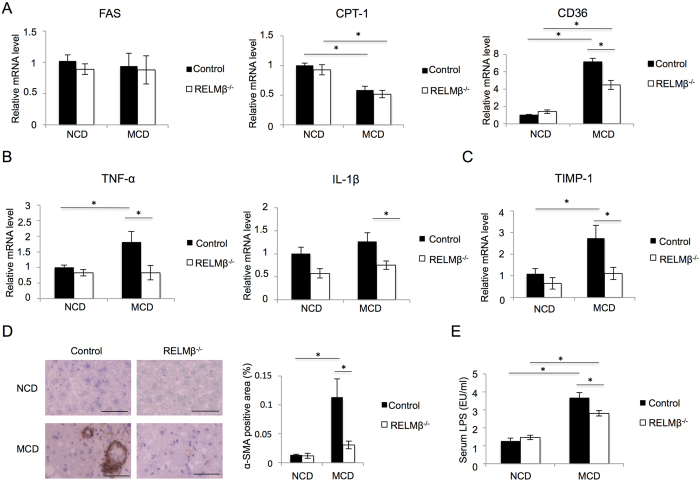
The expressions of genes involved in NASH pathogenesis were suppressed in the livers of RELMβ-KO mice. RELMβ-KO and wild-type mice were fed the NCD (n = 6 per strain) or the MCD diet (n = 6 per strain) for 8 weeks and then sacrificed. (**A**) Hepatic mRNA levels of fatty acid synthase (FAS), carnitine palmitoyltransferase 1 (CPT-1) and CD36 were measured by quantitative real-time PCR (qRT-PCR). (**B**) Hepatic mRNA levels of tumor necrosis factor α (TNF-α) and IL-1β were measured by qRT-PCR. (**C**) The hepatic mRNA level of tissue inhibitor of metalloproteinase 1 (TIMP-1) was measured by qRT-PCR. (**D**) Immunohistochemical staining for α smooth muscle action (α-SMA). Scale bar = 50 μm (×40 magnification). Positively stained areas were counted employing NIH image. (**E**) Serum lipopolysaccharide (LPS) was measured. Data are presented as means ± SE. *Statistical significance P < 0.05.

To investigate the molecular mechanisms underlying the resistance to NASH development of RELMβ-KO mice, we analyzed the hepatic expression levels of factors involved in lipid accumulation, inflammation and fibrotic changes. The mRNA expressions of the lipogenic enzyme fatty acid synthase (FAS) and the β-oxidation enzyme carnitine palmitoyltransferase 1 (CPT-1) did not differ significantly between the livers of RELMβ**–**KO and wild-type mice. In contrast, CD36 mRNA was shown to be markedly upregulated by MCD diet feeding, and this increase was significantly suppressed in the RELMβ-KO as compared with the wild-type mice (Fig. 2A). CD36 reportedly facilitates uptake and intracellular trafficking of free fatty acids, and hepatic CD36 upregulation was shown to be associated with increased steatosis in human NASH^24^. However, taking into consideration that the major source of hepatic lipids is fructose and glucose directly entering the liver via the portal vein in the MCD diet model, the reduced expression of CD36 cannot be regarded as a major mechanism underlying the resistance to liver steatosis in RELMβ-KO mice.

A consensus has been reached that inflammatory cytokines play an important role in the pathogenesis of NASH, and RELMβ reportedly activates macrophages to induce inflammatory cytokine expressions^18^. A previous report suggested hepatic CD36 expression to be induced by inflammatory cytokines^25^. Thus, we investigated and compared hepatic mRNA levels of inflammatory cytokines between RELMβ-KO and wild-type mice. While hepatic TNF-α and IL-1β mRNA levels were upregulated in wild-type mice by MCD diet feeding, these increases were completely prevented in RELMβ-KO mice (Fig. 2B).

Tissue inhibitor of metalloproteinase1 (TIMP-1) expressed in activated hepatic stellate cells (HSC) reportedly plays a critical role in the process of liver fibrosis^26^. TIMP-1 mRNA was also elevated in the livers of wild-type mice by MCD diet feeding, while being only minimally elevated in those of RELMβ-KO mice (Fig. 2C). In accordance with these observations, immunohistochemical analysis also revealed an activated HSC marker, α-smooth muscle actin (α-SMA), to show accumulation in the livers of wild-type mice with MCD diet feeding, while being suppressed in those of RELMβ-KO mice (Fig. 2D). These data suggested HSC activation and liver fibrosis to be suppressed in RELMβ-KO mice.

### The MCD diet-induced increase in serum LPS concentration was suppressed in RELMβ-KO mice

Since the role of endotoxin in the pathogenesis of non-alcoholic fatty liver disease was demonstrated in an animal model^27^, we examined the level of serum LPS as an endotoxin. While MCD diet-feeding markedly elevated serum LPS, this elevation was significantly suppressed in RELMβ-KO mice as compared with wild-type mice (Fig. 2E).

### RELMβ expression levels in the colon and the numbers of RELMβ-positive Kupffer cells were both increased in MCD diet-induced NASH model mice

**Figure 3:**
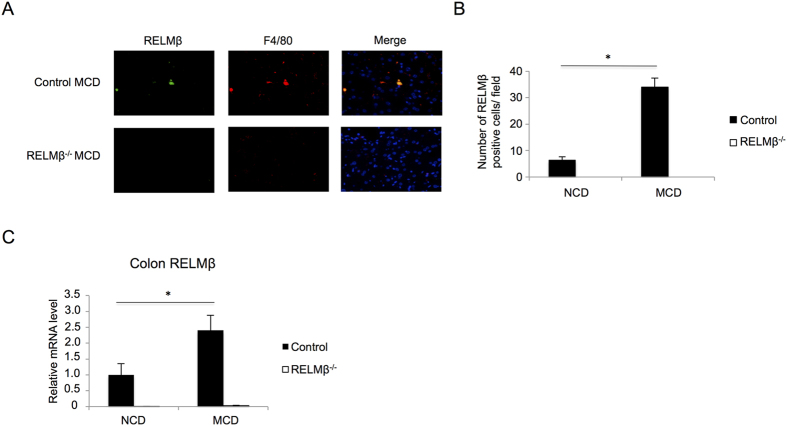
RELMβ expressions in macrophages and the colon were increased by MCD diet feeding. RELMβ-KO and wild-type mice were fed the NCD (n = 6 per strain) or the MCD diet (n = 6 per strain) for 8 weeks and then sacrificed. (**A**) Immunofluorescences of RELMβ (green) and F4/80 (red) in the livers of control and RELMβ-KO mice after MCD diet feeding for 8 weeks are shown. Nuclei were stained with 4-diamidine-2-phenylindole hydrochloride (DAPI; blue). Magnification, ×20. (**B**) Number of RELMβ -positive Kupffer cells in the liver. (**C**) Colon RELMβ mRNA level was measured by quantitative real-time PCR. Data are presented as means ± SE. *Statistical significance P < 0.05.

Immunostaining using anti-RELMβ and F4/80 antibodies was performed on sections of the mouse livers. Interestingly, Fig. 3A shows that RELMβ-positive cells were detected in murine livers, and these cells were identified as Kupffer cells based on being F4/80 positive. Comparison between the RELMβ-KO and wild-type mice fed the NCD or the MCD diet for 8 weeks revealed that the numbers of RELMβ-positive cells were increased by MCD diet feeding, while being undetectable in the livers of RELMβ-KO mice (Fig. 3B). In addition, analysis using real-time PCR revealed the RELMβ mRNA level in the colon to be significantly elevated by MCD diet feeding (Fig. 3C). No RELMβ expression was observed in RELMβ-KO mice. These data indicate that the expression of RELMβ is markedly elevated by MCD diet feeding in both Kupffer cells and the colon.

### Macrophages isolated from RELMβ-KO mice showed reduced responsiveness to LPS

**Figure 4:**
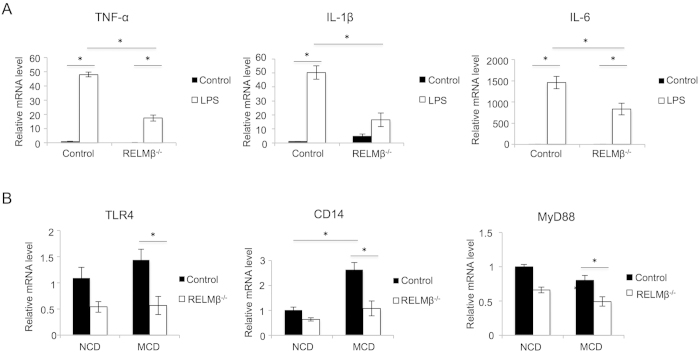
Peritoneal macrophages isolated from RELMβ-KO mice showed attenuated responsiveness to LPS. RELMβ-KO and wild-type mice (n = 3 per strain) were injected with 2 ml of 4% thioglycollate medium 3 days prior to harvest of macrophages by peritoneal lavage. Thioglycollate-elicited macrophages were prepared as the plastic tissue culture plate-adherent population of cells from peritoneal exudate lavage fluid. Macrophages from both groups were allowed to adhere for 16 hours to tissue culture wells, and the cells were then treated with 10 ng/ml LPS, or without LPS as a control, for 4 hours. After LPS stimulation, assay mixtures of all treatments were centrifuged and the cells were harvested and the total cellular RNA was extracted. First-strand cDNAs were then synthesized. (**A**) mRNA levels of TNF-α, IL-1β and IL-6 in macrophages were measured by qRT-PCR. (**B**) RELMβ-KO and wild-type mice were fed the NCD (n = 6 per strain) or the MCD diet (n = 6 per strain) for 8 weeks and then sacrificed. Hepatic mRNA levels of toll-like receptor (TLR) 4, CD14, and MyD88 were measured by qRT-PCR. Data are presented as means ± SE. *Statistical significance P < 0.05.

To examine whether the presence or absence of RELMβ expression affects responsiveness to LPS in terms of cytokine expressions, primary macrophages were isolated from RELMβ-KO and wild-type mice (Fig. 4A). These macrophages were cultured for 24 hours and then stimulated with LPS. Although LPS stimulation increased the mRNA levels of inflammatory cytokines such as TNF-α, IL-1β and IL-6, the degrees of these increases were significantly milder in macrophages from the RELMβ-KO mice. These data suggest that RELMβ enhances the responsiveness of inflammatory cytokine productions to LPS.

### Expression levels of toll-like receptor 4 complex in the liver were suppressed in RELMβ-KO mice fed the MCD diet

LPS stimulates inflammatory cytokine production via toll-like receptor 4 (TLR4) complex expressed on hepatocytes including Kupffer cells, a response considered to play an important role in the pathogenesis of NASH^28^. Thus, we investigated the expression of TLR4 complex components such as the TLR4 co-receptor CD14 and the TLR adaptor protein MyD88 in the mouse liver. Hepatic mRNA levels of TLR4 and MyD88 were suppressed in RELMβ**–**KO as compared with wild-type mice in response to MCD diet feeding. While hepatic CD14 mRNA levels were upregulated in wild-type mice by MCD diet feeding, these increases were suppressed in RELMβ**–**KO mice (Fig. 4B). These data suggest that RELMβ deficiency attenuates the responsiveness of inflammatory cytokine productions to LPS via downregulation of TLR4 signaling.

### Comprehensive analysis of transcriptional changes in macrophages caused by RELMβ

As shown in Fig. 4, RELMβ-KO macrophages exhibit lower responsiveness to LPS stimulation than wild-type macrophages. Thus, to elucidate the regulatory action of LPS stimulation and changes in RELMβ deficiency, we performed a microarray comparison of macrophages from RELMβ-KO mice and their littermates. Table 1 shows the results of the analysis of transcriptional regulation in response to LPS stimulation and changes in accordance with the presence or absence of the RELMβ gene in primary cultured peritoneal macrophages (PCPMs) using KeyMolnet Lite. Transcriptional regulations governed by the extracted genes are presented in ascending order by p value. Effects of LPS are presented in Table 1A: genes showing altered expression levels by more than two-fold with LPS treatment were extracted. Many inflammatory genes showed significant changes and were thus extracted. Nuclear factor κB (NF-κB) and many of the inflammation-regulating transcriptional factors are also listed. Table 1B shows transcriptional factors that regulate genes reducing the effects of LPS stimulation by more than 50% in the setting of RELMβ deficiency. These are transcriptional factors involved in inflammatory regulation such as NF-κB, and lipid accumulation systems such as peroxisome proliferator activated receptor γ (PPARγ). A sky-blue background indicates transcriptional factors with expressions which are significantly impacted by LPS treatment but normalized in the setting of RELMβ deficiency. Differentially expressed genes regulated by NF-kB and PPARγ for each condition are shown in Supplementary Fig. 1A,B. These data suggested that RELMβ deficiency attenuated inflammation in the liver by endotoxins such as LPS.

### Differences between gut microbiota of RELMβ-KO and wild-type mice

**Figure 5:**
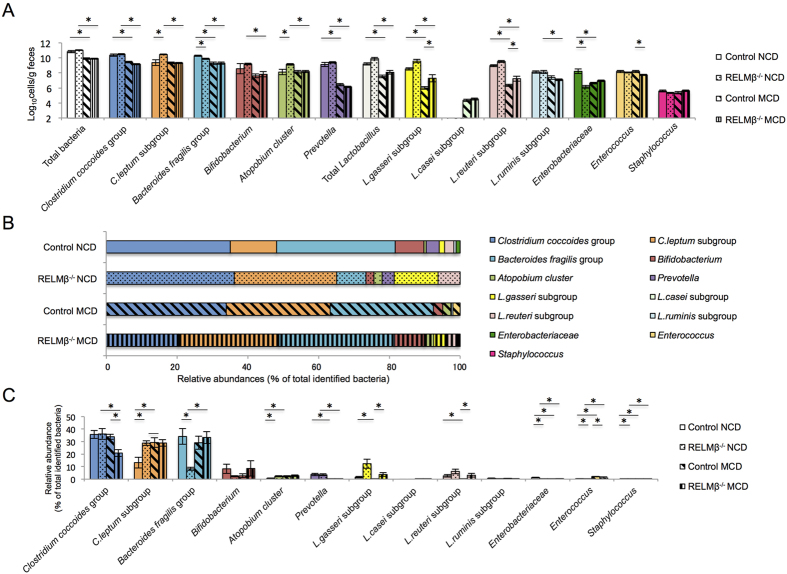
Differences between gut microbiota of RELMβ-KO and wild-type mice. At 5–6 weeks of age, wild-type (n = 12) and RELMβ-KO mice (n = 16) were switched from the NCD to the MCD diet and feeding was continued for 6 weeks. At the start and at the end of the MCD diet feeding, feces were collected and their bacterial contents were analyzed. Numbers of viable bacteria were expressed as cells per gram of feces. (**A**) Bacteria in feces in each group. (**B**) Bar chart of relative abundances of different bacterial species expressed as the percentage of total bacteria. (**C**) Relative abundances of different bacterial species expressed as the percentage of total bacteria. Data are presented as means ± SE. *Statistical significance P < 0.05.

LPS produced in the gut would be absorbed into the blood stream with an efficiency dependent on the barrier function of the gut^29^,^30^. Altered gut microbiota may affect not only the barrier function of the gut but also the amount of LPS produced in the gut. Notably, it was reported that RELMβ deficiency is associated with the gut microbiota composition^14^. Thus, we investigated differences in the gut microbiota of RELMβ-KO and wild-type mice fed the NCD or the MCD diet. As shown in Fig. 5A, gut microbiota differed significantly between the RELMβ-KO and wild-type mice, when fed either the MCD diet or the NCD, and the ratios of each bacterial subgroup to total bacteria are shown in Fig. 5B,C. Interestingly, the proportions of *L. gasseri* subgroup and *L. reuteri* subgroup organisms, when fed the NCD, were higher in the gut microbiota of RELMβ-KO mice than in that of wild-type mice. In addition, after MCD diet feeding for 6 weeks, the proportions of the *L. gasseri* subgroup and *L. reuteri* subgroup organisms belonging to lactic acid bacterial species were significantly higher in RELMβ-KO mice than in wild-type mice, whereas that of the *Clostridium coccoides* group was lower in RELMβ-KO mice than in wild-type mice after MCD diet feeding (Fig. 5C).

### Requirement of both non-hematopoietic and hematopoietic cell-derived RELMβ for full manifestation of NASH

**Figure 6:**
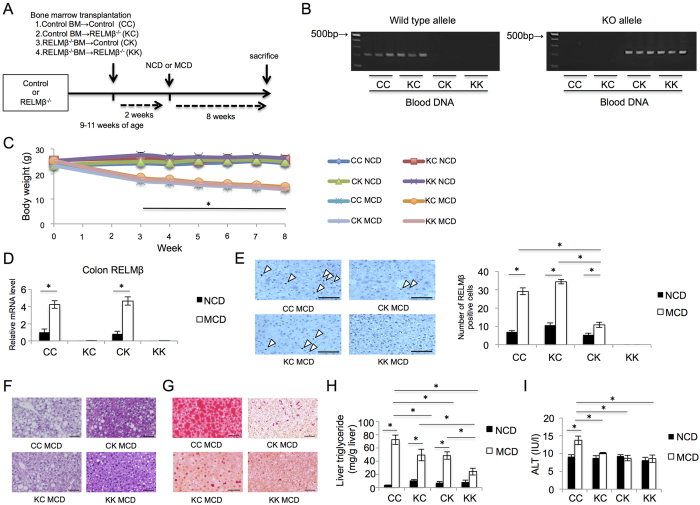
Hematopoietic and non-hematopoietic RELMβ are both involved in the pathogenesis of NASH. Chimeric mice were generated by bone marrow transplantation (BMT). The following four groups were generated: Control BM→Control mice (CC); Control BM→RELMβ-KO mice (KC); RELMβ-KO BM →Control mice (CK); and RELMβ-KO BM →RELMβ-KO mice. After BMT, each group was fed the NCD (n = 6 per strain) or the MCD diet (n = 7 per strain) for 8 weeks and then sacrificed. (**A**) BMT protocol. (**B**) Reconstitution of donor bone marrow was confirmed by PCR. (**C**) Body weight changes. (**D**) The colon RELMβ mRNA level was measured by quantitative real-time PCR. (**E**) Immunohistochemical staining for RELMβ. Number of RELMβ-positive Kupffer cells in the liver. Scale bar = 50 μm (×20 magnification). Arrows indicate RELMβ-positive Kupffer cells. (**F**) Liver sections were stained with HE in the MCD diet fed group. Scale bar = 50 μm (×20 magnification). (**G**) Liver sections were stained with Oil-Red O in the MCD diet fed group. Scale bar = 50 μm (×20 magnification). (**H**) Hepatic triglyceride levels were measured in the NCD and MCD diet fed groups. (**I**) Serum ALT levels were measured in the NCD and MCD diet fed groups. Data are presented as means ± SE. *Statistical significance P < 0.05.

Next, we addressed the question of which RELMβ, that secreted by the colon or that from Kupffer cells, contributes to the pathogenesis of NASH. Bone marrow transplantation (BMT) was carried out between RELMβ-KO and wild-type mice (Fig. 6A). BM from control mice was transplanted into RELMβ-KO mice and vice versa, obtaining mice with RELMβ-sufficient BM-derived cells, including the resident macrophages of the liver; Kupffer cells and RELMβ-KO-deficient endogenous colon cells, and vice versa. As controls, BM from control mice was transplanted into control mice and BM from RELMβ-KO mice was transplanted into RELMβ-KO mice. This strategy produced four different groups of mice (1) Wild-type BM→Wild-type mice (CC), (2) Wild-type BM→RELMβ-KO mice (KC), (3) RELMβ-KO BM→Wild-type mice (CK) and (4) RELMβ-KO BM→RELMβ-KO mice (KK).

After BMT, mice were fed the NCD or the MCD diet for 8 weeks (Fig. 6A). To confirm reconstitution with donor bone marrow, the DNA allele from the blood of recipient mice was checked by PCR (Fig. 6B). There were no significant differences in body weights among the groups with either diet (Fig. 6C). The RELMβ mRNA expression levels in the colon were increased by the MCD diet feeding as compared with NCD in the CC and CK group mice (Fig. 6D). In contrast, there was no RELMβ expression in the colons of KC and KK group mice.

Next, the numbers of RELMβ-positive cells in the livers of mice from the 4 groups were examined (Fig. 6E). MCD diet feeding markedly increased RELMβ-positive cells in the livers of the CC and KC group mice, into which RELMβ-positive BM had been transplanted. In the CK group, RELMβ-positive cells were detected but the numbers were far smaller than in the CC or KC group mice. The survival of some RELMβ-positive cells in the CK group was due to lack of treatment with clodronate prior to radiation. According to a recent report^31^, approximately one-third of Kupffer cells of recipient mice will survive, if clodronate is not used prior to radiation (Fig. 6E), while other hematopoietic cells are completely replaced (data not shown).

HE and Oil-red O staining revealed lipid droplets to be markedly reduced in the livers of mice lacking RELMβ in either non-hematopoietic (CK) or hematopoietic (KC) cells, as compared to mice with RELMβ of both origins (CC), but were still more abundant than in the whole body RELMβ-deficient mice (KK) (Fig. 6F,G). These staining results were in good accordance with the quantitative analysis of liver triglyceride accumulation (Fig. 6H). In addition, MCD diet-induced elevations of serum ALT were suppressed in the mice with neither hematopoietic nor non-hematopoietic RELMβ (Fig. 6I).

**Figure 7:**
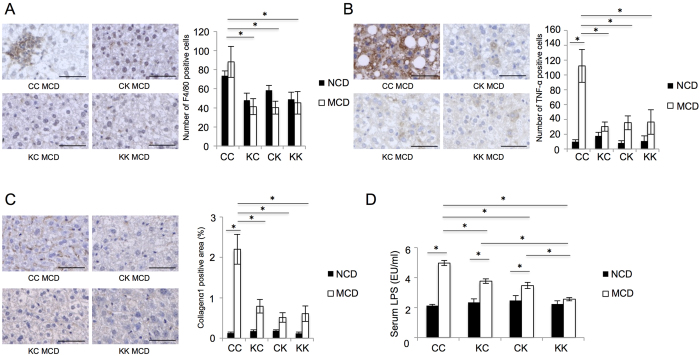
Hematopoietic and non-hematopoietic RELMβ are both necessary for liver inflammation and fibrosis. Chimeric mice were generated by bone marrow transplantation (BMT). The following four groups were generated: Control BM→Control mice (CC); Control BM→RELMβ-KO mice (KC); RELMβ-KO BM →Control mice (CK); and RELMβ-KO BM →RELMβ-KO mice. After BMT, each group was fed the NCD (n = 6 per strain) or the MCD diet (n = 7 per strain) for 8 weeks and then sacrificed. (**A**) Immunohistochemical staining for F4/80 in the NCD and MCD diet fed groups. Scale bar = 50 μm (×40 magnification). Positively stained cells were counted employing NIH image. (**B**) Immunohistochemical staining for tumor necrosis factor α (TNF-α) in the NCD and MCD diet fed groups. Scale bar = 50 μm (×40 magnification). Positively stained cells were counted employing NIH image. (**C**) Immunohistochemical staining for Collagen α1 in the NCD and MCD diet fed groups. Scale bar = 50 μm (×40 magnification). Positively stained areas were counted employing NIH image. (**D**) Serum lipopolysaccharide (LPS) was measured in the NCD and MCD diet fed groups. Data are presented as means ± SE. *Statistical significance P < 0.05.

Subsequently, we carried out immunohistochemical analyses using anti-F4/80, anti-TNF-α and anti-collagen α1 antibodies. The livers from the mice with both non-hematopoietic and hematopoietic RELMβ (CC) showed a higher density of F4/80 positive cells, as compared with the other groups, and there were no significant differences among the CK, KC and KK groups (Fig. 7A). Kupffer cells are the primary source of hepatic inflammatory cytokines, and the numbers of TNF-α positive cells were also examined. In the mouse livers with RELMβ of both origins, TNF-α positive cells were markedly increased by MCD diet feeding, while this increase was significantly suppressed in those with only one or neither RELMβ (Fig. 7B). Very similar results were obtained for the immunostaining of collagen α1, showing the livers with both non-hematopoietic and hematopoietic RELMβ to respond to MCD diet feeding (Fig. 7C). These results suggested that RELMβ secreted from both colonic cells and macrophages augmented macrophage recruitment and inflammatory cytokine expressions and, thereby, promoted liver fibrosis.

### Deficiency of RELMβ secretion from either the colon or macrophages attenuated endotoxemia

Serum LPS concentrations were also examined in the 4 groups receiving BMT. Deficiency of either hematopoietic or non-hematopoietic RELMβ partially suppressed the serum LPS concentration elevations caused by MCD diet feeding. Deficiency of both non-hematopoietic and hematopoietic RELMβ resulted in the lowest LPS concentration among the 4 groups (Fig. 7D). These data raise the possibility that not only RELMβ from the colon but also that secreted by macrophages present in the mucosa of the gut influences serum LPS concentrations.

## Discussion

We previously reported that transgenic mice overexpressing RELMβ showed significant insulin resistance with fatty liver when fed a high fat diet^23^, findings which already suggested the involvement of RELMβ in the pathogenesis of insulin resistance. For this reason, in the present study designed to investigate the contribution of RELMβ to the pathogenesis of NASH, we adopted the MCD diet-induced NASH model which does not show either obesity or insulin resistance in association with NASH. The mechanism underlying the MCD diet-induced NASH development involves impairment of the secretory process of very low-density lipoprotein from the liver, which leads to hepatic lipid accumulation. Although the MCD diet also induces severe inflammation and fibrosis, which are typical of liver cirrhosis, MCD diet-induced mouse phenotypes are different from those of human NASH patients, in terms of body weight loss as well as the absence of insulin resistance in MCD diet-fed mice^32^. Thus, in this study, improvements in adiposity and insulin resistance appeared to play no role in the contribution of RELMβ to the pathogenesis of NASH.

First, we clearly demonstrated RELMβ-KO mice to be highly resistant to MCD-induced NASH, as shown by triglyceride accumulation, fibrotic markers, inflammatory cytokine expressions and immunochemical staining. In addition, importantly, RELMβ expression was shown to be upregulated in the colons of MCD diet-fed mice, and RELMβ-positive Kupffer cells were also increased in the livers of MCD diet-fed mice as compared with NCD-fed mice. Thus, to examine whether the involvement of the RELMβ in the colon or that in Kupffer cells, or both, is critical for NASH development, mice lacking RELMβ in either non-hematopoietic or hematopoietic cells were produced by BMT between RELMβ-KO and wild-type mice. Since clodronate was not used before radiation in our experiments, 30% of RELMβ-positive Kupffer cells in the wild-type mice survived irradiation and subsequent transplantation of RELMβ-KO mouse BM, versus complete absence in the wild-type mice (compare CC and CK mice fed MCD, Fig. 6E), while other hematopoietic cells were entirely replaced (data not shown), which agrees with a previous report^31^. Even considering the survival of some Kupffer cells in this study, the fact that only the mice with both non-hematopoietic and hematopoietic RELMβ developed the full manifestations of NASH (Fig. 6F–I) clearly indicates that both forms of RELMβ have critical roles. In other words, if either hepatic or hematopoietic RELMβ is deleted or suppressed, NASH does not develop.

The mice lacking RELMβ in either non-hematopoietic or hematopoietic cells exhibited a partial reduction in hepatic lipid accumulation (Fig. 6G,H) and also blunting of the elevated serum LPS concentrations induced by MCD diet feeding (Fig. 7D). We speculate that RELMβ derived not only from the intestinal epithelium but also from macrophages immediately below the epithelium might affect gut permeability relating to the serum LPS concentration. Elevated serum LPS appears to lead to hepatic lipid accumulation, which was demonstrated by experiments with LPS infusion into mice^33^.

In addition, besides serum LPS, we consider the RELMβ in Kupffer cells to enhance hepatic inflammation, since LPS-induced inflammatory cytokine expressions were significantly reduced in peritoneal macrophages isolated from RELMβ-KO mice (Fig. 4A) as well as in RELMβ siRNA-treated macrophage cell lines (data not shown), observations in good agreement with those of previous reports showing RELMβ activated macrophages to express MHC class II and pro-inflammatory cytokines^19^,^20^,^21^. It is reasonable to speculate that RELMβ secreted by Kupffer cells induces the expressions of inflammatory cytokines in autocrine and paracrine manners, since the expressions of LPS-induced inflammatory regulation genes located downstream from NFκB were confirmed by microarray comparison of macrophages from RELMβ-KO mice and their littermates (Table 1). Therefore, RELMβ induces not only elevations of serum LPS concentrations but also renders macrophages highly responsive to LPS.

We speculate that elevated saturated fatty acids or TNF-α contents in the liver might trigger RELMβ expression, though the involvement of multiple complex factors including several metabolites or microbes in the gut cannot be ruled out. Indeed, lactic acid bacteria such as *Bifidobacterium* and *Lactobacillus* in feces were markedly reduced by the MCD diet, and RELMβ-KO mice had higher levels of some *Lactobacillus* organisms among gut microbiota (Fig. 5). Some *Lactobacillus* species reportedly contribute to the normalization of tight junction proteins^34^,^35^, and our recent study clearly showed that *Lactobacillus casei* strain Shirota intervention markedly suppressed MCD-diet induced NASH development, with reduced serum LPS concentrations^36^. Taking these observations together, protection against MCD diet-induced impaired gut permeability in RELMβ-KO mice might be partially attributable to this *Lactobacillus* increase, and RELMβ-induced gut microbiota change might be involved in the impairment of gut permeability and the induction of endotoxemia, and thereby in the hepatic inflammation observed in this study. It is also possible that RELMβ itself is a key factor reducing gut barrier function independently of the gut microbiota, although there is one contradictory report suggesting RELMβ to play a beneficial role in maintaining barrier function^20^.

In conclusion, this is the first demonstration of the critical role of RELMβ in the pathogenesis of NASH. Based on our present data, we propose RELMβ to be a possible novel target for NASH therapy. In addition, RELMβ concentrations in serum and/or stool might serve as a marker for assessing the progression of NASH. Taking into consideration the absence of a particularly unfavorable phenotype in RELMβ-KO, research on this approach should be pursued by refining the procedures so as to develop administration methods acceptable for human use.

## Methods

### Animals and treatments

RELMβ-KO mice were generated in collaboration with Lexicon Pharmaceuticals, Inc. (The Woodlands, TX, USA). C57BL/6 mice (SLC, Hamamatsu, Japan) were purchased as controls. Male mice at 5–6 weeks of age from each genotype were fed either the methionine-choline deficient (MCD) diet (Oriental Yeast, Tokyo, Japan) (n = 6, both groups) or a normal chow diet (NCD) (Oriental Yeast, Tokyo, Japan) as the control diet (n = 6, both groups) for 8 weeks. Then, they were killed, and their sera, livers and colons were collected. The animals were handled in accordance with the guidelines for the care and use of experimental animals published by the Japanese Association for Laboratory Animal Science, and animal experiments were carried out in strict accordance with the recommendations in the Guide for the Care and Use of Laboratory Animals of the Hiroshima University Animal Research Committee. All protocols were approved by the Institutional Review Board of Hiroshima University.

### Quantitative real-time PCR

Total RNA was extracted from mouse livers and colons using Sepasol reagent (Nakalai Tesche, Kyoto, Japan). Quantitative real-time PCR (qRT-PCR) was performed using SYBR Green PCR master mix (Invitrogen, Tokyo, Japan) on a CFX96 real time PCR system (Bio-Rad, Tokyo, Japan). Relative mRNA gene levels were normalized to the GAPDH mRNA levels and relative expressions were determined by the comparative *Ct* method. The designed primers were as follows: fatty acid synthase forward (FAS): GCTGCGGAAACTTCAGGAAAT; FAS reverse: AGAGACGTGTCACTCCTGGACTT, carnitine palmitoyltransferase 1 (CPT-1): CCAGGCTACAGTGGGACATT; CPT-1 reverse: GAACTTGCCCATGTCCTTGT, CD36 forward: TGCTGGAGCTGTTATTGGTG; CD36 reverse: TGGGTTTTGCACATCAAAGA, tumor necrosis factor α) (TNF-α) forward: GTAGCCCACGTCGTAGCAAAC; TNF-α reverse: CTGGCACCACTAGTTGGTTGTC, IL-1β forward: TCGCTCAGGGTCACAAGAAA; IL-1β reverse: CATCAGAGGCAAGGCAAGGAGGAAAC, tissue inhibitor of metalloproteinase1 (TIMP-1) forward: ATTCAAGGCTGTGGGAAATG; TIMP-1 reverse: CTCAGAGTACGCCAGGGAAC, RELMβ forward: CAAAAAGCTAGAACTGAGCTCCAG; RELMβ reverse: TAGTAATATGAAGACAATGAGTCAGG, IL-6 forward: CCATCCAGTTGCCTTCTTGG; IL-6 reverse: TCCACGATTTCCCAGAGAACA, toll-like receptor 4 (TLR4) forward: GCCTTTCAGGGAATTAAGCTCC; TLR4 reverse: AGATCAACCGATGGACGTGTAA, CD14 forward: GAGTTGTGACTGGCCCAGTCAGC; CD14 reverse: GCAAAAGCCAGAGTTCCTGAC, MyD88 forward: AGAACAGACAGACTATCGGCT; MyD88 reverse: CGGCGACACCTTTTCTCAAT.

### Immunohistochemical analysis

Paraffin-embedded sections from mouse livers were stained with hematoxylin and eosin (HE). Histological evaluations were performed employing the histological scoring system for NAFLD^37^. Oil Red-O staining was performed on frozen liver sections. Immunohistochemical staining with anti-Collagen α 1 antibody (Abcam, Cambridge, MA, USA), anti-αSMA antibody (Abcam), anti-F4/80 antibody (Abcam), or anti-TNF-α antibody (Abcam) was performed by SRL Co. Ltd (Tokyo, Japan). Immunohistochemical staining with mouse RELMβ of the liver sections was performed using anti-mRELMβ antibody (Abcam), and simple stain mouse MAX-PO (R) (Nichirei, Tokyo, Japan) was used as the secondary antibody. Immunofluorescence staining was performed using rat anti-mouse F4/80 antibody (Serotec, Oxford, UK) and anti-mRELMβ antibody. Alexa-Fluor 546 and 488 (Invitrogen, CA, USA) were used as the secondary antibodies. Digital images of lesions were randomly selected and positive cells were counted using a multifunctional microscope (BZ-9000; KEYENCE Co, Osaka, Japan), Image-J (National Institute of Health, MD, USA) or FSX 100 Olympus Microscope (Olympus America Inc., Center Valley, PA).

### Biochemical analysis

Serum alanine aminotransferase (ALT) activity was determined using a Transaminase C-II test kit (Wako, Osaka, Japan). Hepatic total lipid was extracted and then assayed using the Folch method^38^. The triglyceride content was assayed with the Triglyceride E test by Wako (Wako, Osaka, Japan). Serum lipopolysaccharide (LPS) concentrations were determined using the LAL kit endpoint QCL-1000 (Walkersville, MD, USA), according to the manufacturer’s instructions.

### Stimulation of primary-cultured macrophages with LPS

Control mice or RELMβ-KO mice were injected with 2 ml of 4% thioglycollate medium 3 days prior to harvest of macrophages by peritoneal lavage. Thioglycollate-elicited macrophages were prepared as the plastic tissue culture plate-adherent population of cells from peritoneal exudate lavage fluid. Macrophages from both groups were allowed to adhere for 16 hours to tissue culture wells, and the cells were then treated with or without 10 ng/ml LPS (Sigma, St. Louis, MO, USA) for 4 hours. After LPS stimulation, assay mixtures of all treatments were centrifuged at 2000 rpm for 20 min. The cells were harvested and the total cellular RNA was extracted as described above. Then, first-strand cDNAs were synthesized and quantitative real-time PCR (qRT-PCR) was performed as described above.

### Microarray comparison of macrophages from RELMβ-KO and wild-type mice

Primary cultured peritoneal macrophages (PCPMs) were obtained from 3-month-old RELMβ-KO and wild-type mice and cultured for 24 hours as previously described^39^. After 12-hour serum starvation, 10 ng/ml LPS was added and incubation was continued for 4 hours. Then, total RNA was extracted from PCPMs, using Trizol, followed by the RNeasy kit (Qiagen, Crawley, UK). Five micrograms of RNA were subjected to reverse transcription using Transcriptor Reverse Transcriptase (Roche) and hybridization onto Affymetrix MG-430 2.0 microarray chipsets (Affymetrix, CA, USA). The arrays were scanned using the Affymetrix GeneChip Scanner 3000 7G controlled by GeneChip Operating Software, 1.3. Comparisons were performed between no treatment and LPS stimulation as follows; first, genes were extracted from the data using the KeyMolnet Lite ver. 4.6 (IMMD Co.) database. Next, genes showing altered expression levels by more than two-fold with LPS treatment were identified. Then, the transcription factors associated with expressions of these genes were analyzed using KeyMolnet Lite. Other transcriptional factor analyses were performed by comparing these genes between RELMβ-KO and wild-type macrophages.

Genes reducing the effects of LPS stimulation by more than 50% in the setting of RELMβ deficiency were extracted. Then, transcriptional factors regulating these genes were analyzed. Another analysis focused on the pathophysiological events derived from the extracted genes, those for which the stimulatory effects of LPS on their expressions were blunted by RELMβ deficiency. KeyMolnet software was used to calculate the probability of associations between these genes and transcriptional factors.

The p values were calculated employing the following equations, from probability based on a hypergeometric distribution.


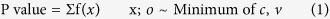



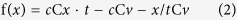


*o*, *t*, *c* and *v* were as follows;*o* was the number of overlaps between disease-mediating molecules and resultant genes. *t* was the total number of molecules in KeyMolnet Lite. *c* was the number of disease-mediating molecules. *v* was the number of resultant genes. The transcriptional factors were listed in order of P values. Values of P < 0.05 were considered to indicate statistically significant differences. To minimize nonessential variance, signals were averaged based on several probes for one gene, with omission of probes indicated to be “absent” in one or more experiments using GeneChip Operating Software.

The fold enrichment scores were calculated employing the following equation.


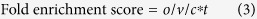


*o*, *t*, *c* and *v* were as follows:*o* was the number of overlaps between molecules regulated by the transcriptional factor and resultant items, indicating altered expression by more than 1.5-fold. *t* was the total number of molecules in KeyMolnet Lite. *c* was the number of molecules regulated by the transcriptional factor. *v* was the number of resultant items.

### Bone marrow transplantation

Femurs of donor mice were flushed to obtain bone marrow (BM). BM cells were injected into the tail veins of lethally irradiated (9.5 Gy) recipient male mice at 9–11 weeks of age. BM from control mice was transplanted into RELMβ-KO mice and vice versa. As controls, BM from control mice was transplanted into control mice and BM from RELMβ-KO mice was transplanted into RELMβ**–**KO mice. After BM transplantation (BMT), each of the four groups was fed either the NCD (n = 6 per group) or the MCD diet (n = 7 per group) for 8 weeks. Reconstitutions with donor bone marrow were confirmed by PCR using Ex TaqDNA polymerase (Takara, Tokyo, Japan). For detection of the wild-type allele, primer29 (TGAAACCACGGTCTCGACC) and primer30 (CCTATCTTTCTTCACCACCC) were used. The RELMβ-KO allele was detected by a set comprised of primer30/primerNeo3A (TGAAACCACGGTCTCGACC)^10^. PCR was performed at 94 °C for 2 min, followed by 35 cycles of amplification (94 °C for 30 sec, 63 °C for 30 sec, and 72 °C for 45 sec) and 72 °C for 10 min.

### Gut microbiota analysis

Control and RELMβ-KO mice were bred in the same cage and kept in the same environment. At 5–6 weeks of age, control and RELMβ-KO mice were switched from the NCD to the MCD diet and feeding was continued for 6 weeks. At the start and at the end of the MCD diet feeding, we collected the feces and bacterial contents were analyzed by 16S rRNA-targeted RT–quantitative PCR using the Yakult Intestinal Flora-SCAN (YIF-SCAN^®^) located at the Yakult Central Institute (Tokyo, Japan), as previously reported^40^,^41^.

### Statistical analysis

Results are expressed as means ± S.E and significance was assessed using ANOVA followed by the Tukey HSD test, unless otherwise indicated.

## Additional Information

**How to cite this article**: Okubo, H. *et al.* Involvement of resistin-like molecule β in the development of methionine-choline deficient diet-induced non-alcoholic steatohepatitis in mice. *Sci. Rep.* **6**, 20157; doi: 10.1038/srep20157 (2016).

## Supplementary Material

Supplementary Information

## Figures and Tables

**Table 1:**
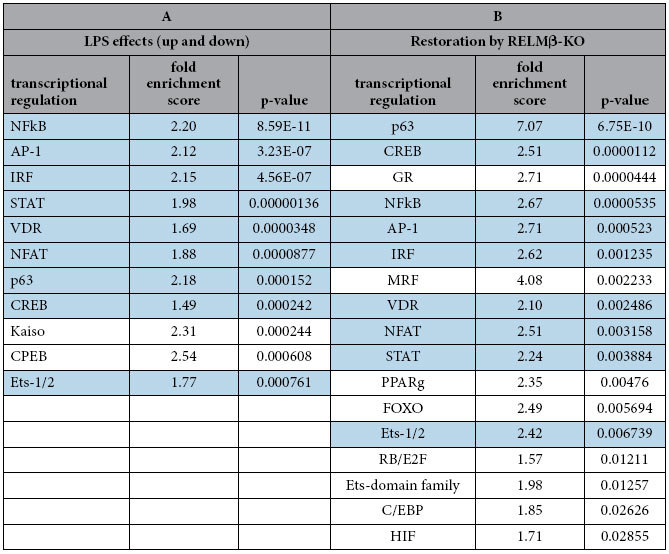
Analysis of the transcriptional regulation mediated by LPS stimulation and regulations associated with RELMβ deficiency using KeyMolnet Lite.

PCPMs were obtained from 3-month-old RELMβ-KO and wild-type mice. After 12-hour serum starvation, 10 ng/ml LPS was added and incubation was continued for 4 hours. Total RNA was extracted from PCPMs and 5 μg of RNA were then subjected to reverse transcription using Transcriptor Reverse Transcriptase (Roche) and hybridization onto Affymetrix MG-430 2.0 microarray chipsets (Affymetrix, CA, USA). A: The following transcriptional factors were used for comparisons between no treatment and LPS stimulation. First, genes were extracted from the data using the KeyMolnet Lite ver. 4.6 (IMMD Co.) database. Next, genes showing altered expression levels by more than two-fold or less than 50% in response to LPS treatment were identified. Then, the transcription factors associated with expressions of these genes were analyzed using KeyMolnet Lite. The P values and fold enrichment scores were calculated employing the equations described in Methods. Values of P < 0.05 were considered to indicate statistically significant differences. B: Genes from RELMβ-KO and wild-type mouse PCPMs were compared. Genes were extracted if the effect of LPS stimulation on their expressions was reduced by more than 50% in the setting of RELMβ deficiency. Then, transcriptional factors regulating these genes were analyzed, and listed in order of P values. A sky-blue background indicates transcriptional factors with expressions significantly impacted by LPS treatment but normalized in the setting of RELMβ deficiency. P < 0.05 was considered to indicate a statistically significant difference.
