# Supplementary material for: Involvement of resistin-like molecule β in the development of methionine-choline deficient diet-induced non-alcoholic steatohepatitis in mice
Source: Sci Rep. 2016 Jan 28;6:20157. doi: 10.1038/srep20157 (PMC4730188; doi:10.1038/srep20157)
Supplement: Supplementary Information [file srep20157-s1.pdf]

Supplementary information

Involvement of resistin-like molecule β in the development of methionine-choline deficient diet-induced non-alcoholic steatohepatitis in mice

Hirofumi Okubo, Akifumi Kushiyama, Hideyuki Sakoda, Yusuke Nakatsu, Masaki Iizuka, Naoyuki Taki, Midori Fujishiro, Toshiaki Fukushima, Hideaki Kamata, Akiko Nagamachi, Toshiya Inaba, Fusanori Nishimura, Hideki Katagiri, Takashi Asahara, Yasuto Yoshida, Osamu Chonan, Jeffery Encinas and Tomoichiro Asano

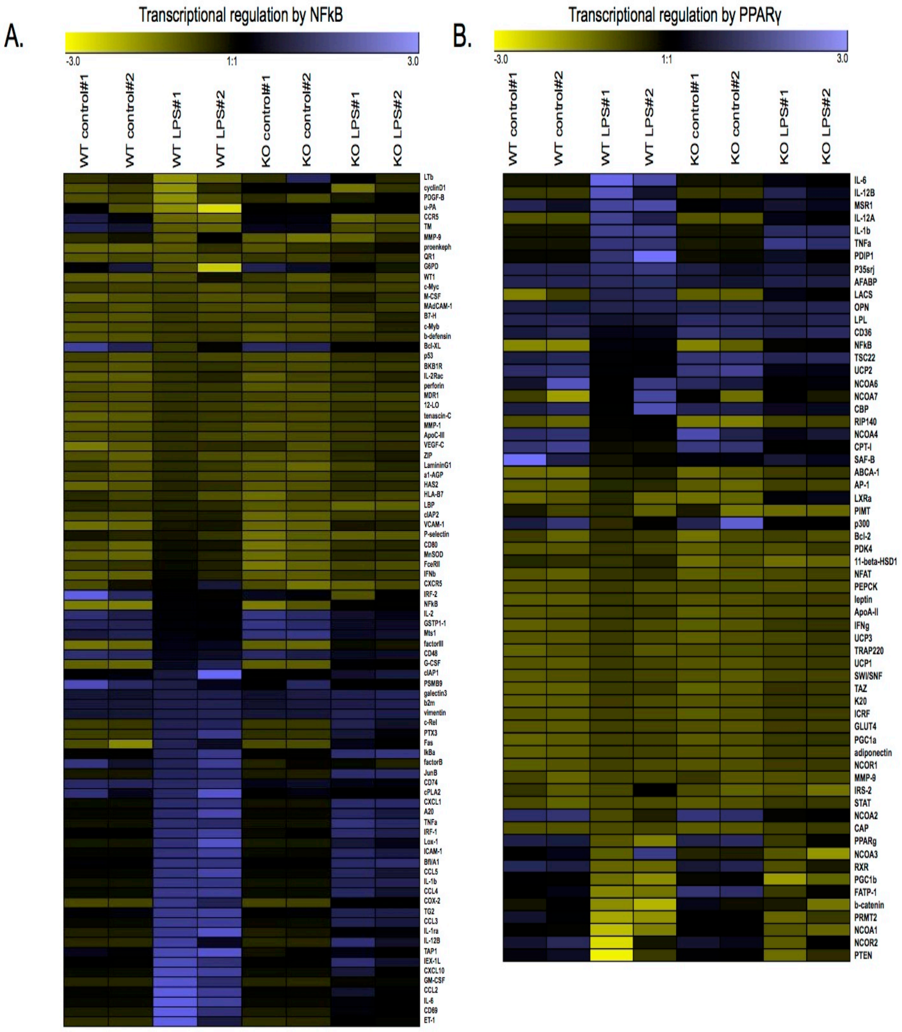

Supplementary Figure 1.

Comprehensive analysis of the expression changes of genes regulated by NFkB and PPARγ in macrophages caused by RELMβ.

PCPMs were obtained from 3-month-old RELMβ-KO and wild-type mice. After 12-hour serum starvation, LPS at 10 ng/ml was added and incubation was continued for 4 hours. Total RNA was extracted from PCPMs and 5 μg of RNA were then subjected to reverse transcription using Transcriptor Reverse Transcriptase (Roche) and hybridization onto Affymetrix MG-430 2.0 microarray chipsets (Affymetrix, CA, USA).

Analysis of the expression changes of genes regulated by NFkB and PPARγ, in response to LPS stimulation, and regulations associated with RELMβ deficiency using KeyMolnet Lite.

A. Heat-map presentation of differentially expressed genes transcriptionally regulated by NFkB in PCPMs obtained from 3-month old RELMβ-KO and wild-type mice with or without LPS treatment.

B. Heat-map presentation of differentially expressed genes transcriptionally regulated by PPARγ in PCPMs obtained from 3-month old RELMβ-KO and wild-type mice with or without LPS treatment.

Blue, downregulated genes; yellow, upregulated genes.
